# On resin click-chemistry-mediated synthesis of novel enkephalin analogues with potent anti-nociceptive activity

**DOI:** 10.1038/s41598-019-42289-5

**Published:** 2019-04-08

**Authors:** Azzurra Stefanucci, Wei Lei, Stefano Pieretti, Ettore Novellino, Marilisa Pia Dimmito, Francesca Marzoli, John M. Streicher, Adriano Mollica

**Affiliations:** 1 Dipartimento di Farmacia, Università di Chieti-Pescara “G. d’Annunzio”, Via dei Vestini 31, 66100 Chieti, Italy; 2 Department of Pharmacology, College of Medicine, University of Arizona, Tucson, AZ USA; 3 Istituto Superiore di Sanità, Centro Nazionale Ricerca e Valutazione Preclinica e Clinica dei farmaci, Viale Regina Elena 299, 00161 Rome, Italy; 4 Dipartimento di Farmacia, Università di Napoli “Federico II”, Via D. Montesano 49, 80131 Naples, Italy

## Abstract

Here, we report the chemical synthesis of two DPDPE analogues **7a** (**NOVA1)** and **7b** (**NOVA2**). This entailed the solid-phase synthesis of two enkephalin precursor chains followed by a Cu^I^-catalyzed azide-alkyne cycloaddition, with the aim of improving *in vivo* analgesic efficacy versus DPDPE. **NOVA2** showed good affinity and selectivity for the μ-opioid receptor (K_I_ of 59.2 nM, EC_50_ of 12.9 nM, E_Max_ of 87.3%), and long lasting anti-nociceptive effects in mice when compared to DPDPE.

## Introduction

DPDPE ((D-Pen^2^,D-Pen^5^)-Enkephalin) represents one of the most successful designed cyclic opioid peptides. It is widely used as a radiolabeled standard for *in vitro* binding assays and as a standard highly selective δ-opioid receptor agonist (DOP)^1,2^. This selectivity is mainly due to its rigid structure, caused by a disulphide bridge and the presence of two D-Penicillamine (DPen) residues together with the *C*-terminus free carboxylic group; in contrast, the disulphide bridge between the two D-Cysteines or a mix of Cysteine and DPen, and the *C*-terminal amide, returns less DOP selectivity and activity^3,4^. However, this importance as a standard drug hasn’t translated to therapeutic application due to a lack of activity when given peripherally, due to intrinsic metabolic instability and low blood brain barrier penetration^5^.

Cyclization of peptides represents a powerful tool to overcome these drawbacks in the fields of drug discovery and development^6–9^. Cyclic peptides are usually obtained by standard techniques such as the formation of amide, ester, disulphide, olefin and C-C bonds, and are abundant and well documented in the literature^10^. Elegant studies employing ring closing metathesis for the cyclization of DPDPE have been published by Mollica *et al*.^11^. and by Schiller *et al*.^12^. Cyclic peptidomimetics are usually characterized by: (*i*) folding in a rigid structure^13^; (*ii*) an improved metabolic stability compared to the linear counterparts^14^; (*iii*) better penetration of the blood-brain barrier (BBB)^15^; (*iv*) improved oral bioavailability and rapid excretion^16^; (*v*) and a better pharmacological profile in terms of potency and selectivity^17^. These features could make DPDPE and other analogues good candidates as leads for drug development, as peptides are generally characterized by high selectivity and low toxicity^18^.

**Figure 1:**
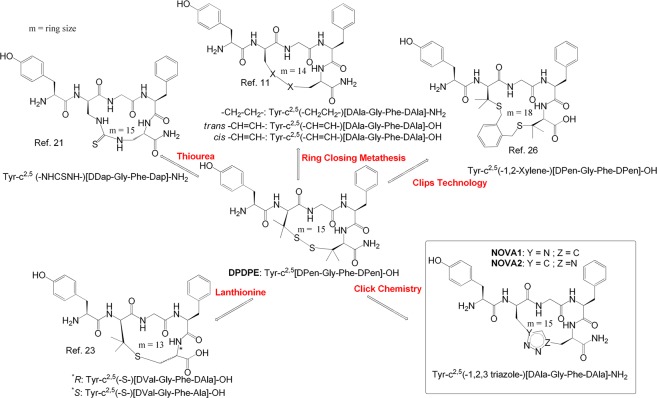
Main structural modifications applied to the cyclic opioid peptide DPDPE.

A great number of works in the literature report the cyclization of enkephalin by diverse approaches (Fig. 1). The 18-membered enkephalin (ENK) analogue cyclo(*N*^ε^,*N*^β’^-carbonyl-D-Lys^2^,Dap^5^)-enkephalinamide was obtained performing the on-resin cyclization reaction between the unprotected side chains of D-Lys and Dap with bis-(4-nitrophenyl)carbonate in the linear peptide sequence, to incorporate the urea moiety^19^. This compound showed high MOP and DOP agonist activities (IC_50_ = 0.21 and 0.65 nM in the GPI and MVD assay, respectively) compared to Leu-ENK, without exhibiting substantial selectivity^19,20^. *N*-terminal amidated ENK analogues containing a substituted guanidine or thiourea bridge gave a 15–22 membered ring depending on the amino acid substitution and positions; the thiourea bridge in the small ring size series resulted in a very potent MOP/DOP agonist (IC_50_ = 1.8 and 2.4 nM in the GPI and MVD assays)^21^. Analogues incorporating the bulky Bcp residue in place of Tyr^1^ of the non-selective cyclic ENK analogue Tyr-c^2,5^[DCys-Gly-Phe(4-NO_2_)-DCys]-NH_2_, DALDA, Tyr-DArg-Phe-Lys-NH_2_, and KOP selective analogue Dyn A(1–11)-NH_2_ retained high MOR affinity, but showed very different receptor selectivity compared to parent analogues^22^. ENK dicarba analogues showed well-rounded MOP/DOP agonist activity^4^, while the presence of the free acid improved selectivity for the DOP over the MOP^11^.

High affinity and selectivity for the DOP was accomplished by using a lanthionine bridge in the scaffold of Tyr-c^2,5^[DAla/Val-Gly-Phe-D/LAla]-OH^23^. The analogue cyclized between DVal^2^ and L/DAla^5^ produced subnanomolar analgesic potencies (ED_50_ = 0.12, 0.26 nM) *in vivo*, which may be due to potent DOP activity and improved stability. All lanthionine-bridged compounds had significantly lower antinociceptive ED_50_ values compared to DPDPE, but increased potency relative to morphine following spinal delivery. The *N*-ureidoethylamides Tyr-c^2,4^[DLys-Phe-Dab]-CH_2_CH_2_NHCONH_2_ showed a stronger antinociceptive response than that of morphine and was resistant to enzymatic degradation^24^.

DPDPE is cleared primarily by biliary excretion, thus *N*-terminal PEGylation decreases hepatic clearance and enhances analgesia following i.v. administration. This PEGylated derivative appears to function as a prodrug, undergoing hydrolysis to the parent peptide^25^. Recently our research group described three analogues of DPDPE containing a xylene bridge in place of the disulphide bond, characterized by different ring sizes^26^. These cyclic peptides possess good *in vitro* affinity for MOP and DOP and one of them is a potent analgesic compound able to maintain a high level of antinociception following intracerebroventricular (i.c.v.) and subcutaneous administration *in vivo*. In contrast, DPDPE was slightly active until 45 min after i.c.v. administration, and completely inactive after s.c. administration^26^.

Prompted by these findings, in the present work we achieved the solid-phase peptide synthesis (SPPS) of two novel cyclic enkephalin analogues, namely **NOVA1** and **NOVA2**, by on-resin Cu^I^-catalyzed 1,3-dipolar cycloaddition of azides and alkynes (CuAAC). We aimed to explore the biological profile imparted by the incorporation of a triazole bridge^27^. The unnatural amino acids D-Propargylglycine (DPra) and D-β-azidoalanine (DAza) were inserted in position 2 and 5 for peptide **NOVA1** and in position 5 and 2 for peptide **NOVA2**, respectively, similar to the DPen residues present in DPDPE. Specifically, DPra was introduced to provide the alkyne functional group, whereas the azide moiety was supplied by the DAza amino acid. The novel cyclic compounds were prepared as *C*-terminal amides according to the findings of Schiller and co-workers^4^. Cu^I^-catalyzed 1,3-dipolar cycloaddition of azides and alkynes “click chemistry” is a biorthogonal reaction, which leads to the formation of 1,4-disubstituted 1,2,3-triazoles^27^. Recently, this reaction has become widely used in organic, medicinal and peptide chemistry, as 1,2,3-triazole is a motif with the structural and electronic characteristics similar to those of the peptide bond^28,29^.

We used a Cu^I^-catalyzed variant of the Huisgen 1,3-dipolar cyclization of azides and alkynes, namely CuAAC, to give 1,2,3-triazole bioactive peptides with high efficiency, due to reaction reliability, biocompatibility, and regioselectivity^30^. The main point of interest in this reaction is the possibility to build the 1,2,3-triazole ring; this attractive connecting unit is stable to metabolic degradation, is capable of hydrogen bonding with biomolecular targets, and can improve solubility^31–34^. The triazole ring has N^2^ and N^3^ nitrogen atoms that are potential hydrogen bond acceptors; the ring itself presents a large dipole alignable with that of the peptide secondary structure’s amides^35^. In addition, triazole units strategically incorporated into the peptide sequence can promote the formation of β-sheet structures and act as α-helical units, an important feature to address complex targets such as DNA and RNA^36,37^.

Four linear analogues of Leu-enkephalin bearing a triazole in different positions of the peptide sequence were prepared using Cu(I)-catalyzed azide-alkyne cycloaddition, demonstrating that the presence of the triazole between Phe and Leu preserves DOP activity and key interactions with the MOP^38^. A series of novel fluorinated triazole containing peptides were also recently identified as myelin-imaging agents able to penetrate the BBB and specifically bind to myelin membranes in the brain and spinal cord^39^. All together these successful reports on the design of macrocycles and small cyclic building blocks containing the triazole moiety^40,41^ prompted us to apply SPPS/CuAAC on resin combination to the synthesis of novel cyclic enkephalin analogues. The biological functionality aspect of this work was focused on the role and modifications of the disulphide bridge between the two DPen residues of DPDPE. The presence of the triazole moiety is expected to improve the biological profile and metabolic stability of the novel chemical entities.

## Results and Discussion

### Chemistry

**Figure 2:**
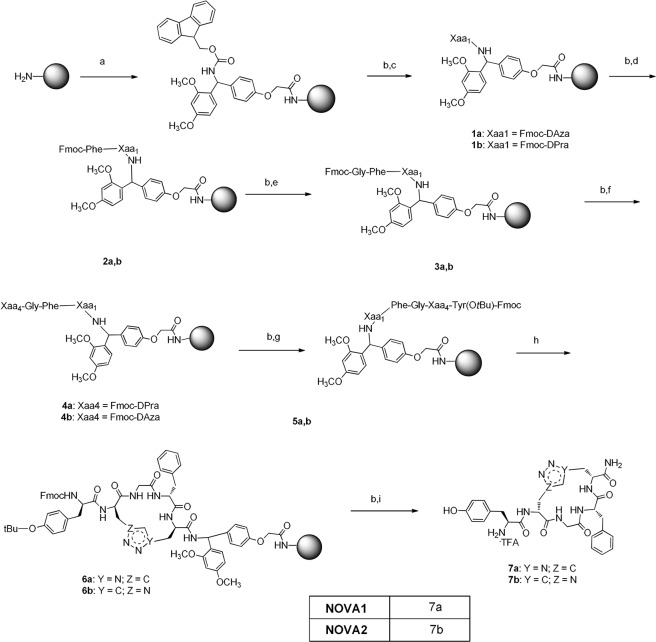
Reagents and conditions (**a**) Fmoc-Rink amide-linker, TBTU, HOBt anhydrous, DIPEA, DMF, at r.t., 24 h. (**b**) piperidine 20% in DMF at r.t. 30 min. (**c**) Fmoc-DAza-OH, HATU, DIPEA, DMF at r.t. or Fmoc-DPra-OH, HATU, DIPEA, DMF at r.t. (**d**) Fmoc-Phe-OH, TBTU, HOBt a., DIPEA, DMF at r.t. (**e**) Fmoc-Gly-OH, TBTU, HOBt a., DIPEA, DMF at r.t. (**f**) Fmoc-DAza-OH, HATU, DIPEA, DMF at r.t. or Fmoc-DPra-OH, HATU, DIPEA, DMF at r.t. (**g**) Fmoc-Tyr(O*t*Bu)-OH, TBTU, HOBt a., DIPEA, DMF at r.t. (**h**) CuBr, sodium ascorbate, water, 2,6-Lutidine, DIPEA, DMSO anhydrous at r.t., N_2_ atmosphere, 24 h. (**i**) TFA:TIS:DCM:water = 90%/2.5%/5%/2.5% at r.t. 2 h.

The novel peptides were prepared following Fmoc-standard SPPS (Fmoc-SPPS) on a polystyrene resin (Tentagel-S-NH_2_ resin, 0.26 mmol.g^−1^ loading) at 0.1-mmol scales, which was functionalized with Fmoc-Rink amide linker, in the presence of TBTU, HOBt anhydrous, and DIPEA in DMF (Fig. 2). A capping procedure was applied to cover the unprotected NH_2_ groups with acetic anhydride/DIPEA/DCM = 1:1:5. The protecting group was removed with a solution of 20% piperidine in DMF and treated with a coupling mixture of the first amino acid (Fmoc-DPra-OH and Fmoc-DAza-OH respectively for **7b** and **7a**), HATU, DIPEA in DMF at r.t. for 24 h. The Kaiser test was used to check the completeness of each reaction. Repeated cycles of coupling reaction/deprotection were performed to reach the complete sequence.

To test the completeness of the **NOVA1** peptide sequence, 1/3 of the dried resin was transferred to a plastic vessel and treated with a cleavage cocktail, then the peptide was precipitated and washed twice with diethyl ether. The so obtained crude product was identified with UPLC-MS and ^1^H-NMR to confirm the peptide linear structure (see Fig. S1 in SI). Then on resin *side chain to side chain* cyclization was performed on the rest of the resin employing CuACC, following the procedure reported by Ingale and Dawson^42^.

The click reaction was performed under N_2_ atmosphere at r.t. for 24 h, then the resin was washed with isopropanol:DMSO = 5:3, DMF and DCM, and the Fmoc protecting group was removed from the peptide *N*-terminus. The crude peptide was cleaved from the resin with a mixture of TFA:TIS:DCM:water = 90%/2.5%/5%/2.5%, precipitated with cold ether and centrifuged to yield a white solid product. A mixture of crude cyclic peptide **7a** and its linear precursor was examined in UPLC-MS to confirm their different identities (Fig. S2, SI). The crude peptides were purified on RP-HPLC to afford the desired products **NOVA1** and **NOVA2** as TFA salts with 15% and 12% yields respectively and ≥95% purity (Figs S3, S4 with validating MS data, see SI). The identity of final peptides was also confirmed by HRMS (Figs S5, S6, see SI) and ^1^H-NMR (Table S1, Figs S7–S10, see SI).

### Opioid receptor binding affinity

**Figure 3:**
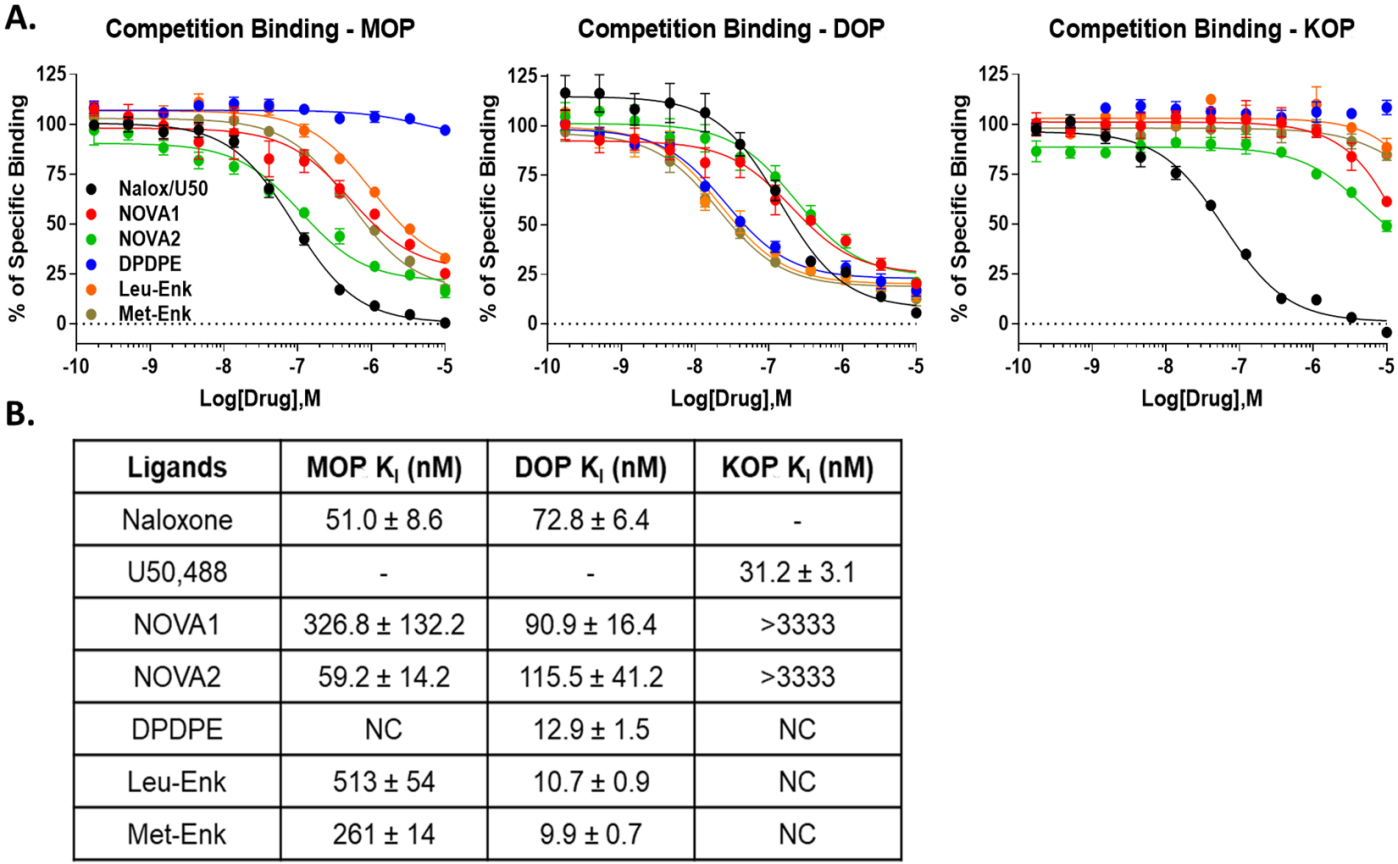
NOVA compounds preferentially bind to the MOP and DOP. (**A**) Competition binding curves for the NOVA compounds and naloxone (MOP, DOP) or U50,488 (KOP) positive controls shown, along with the scaffold/parent controls DPDPE, Leu-Enkephalin, and Met-Enkephalin. All compounds were competed against a fixed concentration of ^3^H-diprenorphine in the membranes of MOP, DOP, or KOP-expressing CHO cells. Summary curves shown with the mean ± SEM of each point from n = 3 independent experiments. (**B**) The K_I_ of each compound was calculated from the IC_50_ of each curve using the previously established K_D_ of ^3^H-diprenorphine in each cell line. Each K_i_ value was calculated from each independent experiment and reported here as the mean ± SEM (n = 3 independent experiments).

The novel cyclic peptides were first tested for their binding affinity at the MOP, DOP, and KOP. The compound **NOVA2** displayed moderate affinity for the MOP (59.2 nM), which was reduced for **NOVA1** (326.8 nM, Fig. 3). Both compounds displayed similar modest affinity for the DOP (~100 nM). Interestingly, both compounds showed very low affinity for the KOP (>3333 nM). The positive control compounds showed expected affinity at all 3 receptors, validating the assay (Fig. 3). In comparison to previously published DPDPE analogues incorporating the xylene moiety^26^, our modifications provided a balanced profile and selectivity for the MOP and DOP, while demonstrating very little KOP binding. These findings provide further SAR information relating to our compound modifications to the cyclic enkephalin structures. Further supporting this conclusion, we found that the parent compounds DPDPE, Leu-Enkephalin, and Met-Enkephalin all showed a DOP-selective binding profile in our hands, versus the more balanced MOP/DOP profile of **NOVA1/2** (Fig. 3).

### Opioid receptor functional activity

**Figure 4:**
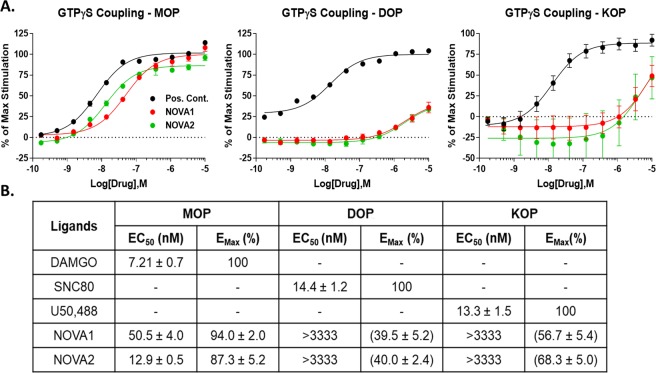
NOVA compounds selectively activate the MOP in the ^35^S-GTPγS coupling assay. (**A**) Summary agonist concentration curves for the NOVA compounds and a positive control agonist (DAMGO for MOP; SNC80 for DOP; U50,488 for KOP) in ^35^S-GTPγS coupling shown. All compounds were normalized to the stimulation caused by the positive control (100%) and vehicle (0%) and reported as the mean ± SEM from n = 3 independent experiments. (**B**) The potencies (EC_50_) and efficacies (E_Max_) of each NOVA compound and positive control were calculated in each independent experiment (n = 3), then reported as the mean ± SEM. The E_Max_ was reported as a percent of stimulation caused by the positive control, which had a defined E_Max_ of 100%. For the DOP and KOP, E_Max_ values in parentheses report the maximum stimulation at 10 μM of compound, not the fully defined top of the agonist curve, as these curves are incomplete at 10 μM.

Both compounds were next evaluated for their ability to activate the MOP, DOP, and KOP using a ^35^S-GTPγS coupling assay. Both **NOVA** compounds activated the MOP with high efficacy (87.3–94.0% vs. DAMGO control, Fig. 4), and an improved 5–6 fold potency vs. the binding affinity of each compound from Fig. 3. This suggests that both compounds possess high intrinsic efficacy at the MOP, and furthermore, that **NOVA2** displays high potency and efficacy MOP agonist activity (EC_50_ of 12.9 nM; E_Max_ of 87.3%, Fig. 4). Intriguingly, both compounds displayed very weak potency agonist activity at the DOP (>3333 nM) while demonstrating reasonable affinity in Fig. 3 (~100 nM). Several possibilities could explain these results. Both **NOVA** compounds could have very weak intrinsic efficacy at the DOP, leading to weak functional potency. Both compounds could also be strongly biased against G protein activation, and could be activating other signaling pathways with greater potency^43^. Both **NOVA** compounds displayed very low potency KOP agonism (>3333 nM, Fig. 4), matching the very low KOP affinity observed in Fig. 3. These results confirm and extend the binding findings, in which the **NOVA** compounds have improved selectivity and activity for the MOP vs. the parent DPDPE structure. These cyclic peptides containing a triazole moiety preserve a good MOP/DOP affinity as the previously described analogues incorporating a xylene bridge^26^, but lost the DOP selectivity of DPDPE and surprisingly act as selective MOP agonists unlike the urea and thiourea containing ENK cyclic analogues^20,21^.

### Nociception tests

**Figure 5:**
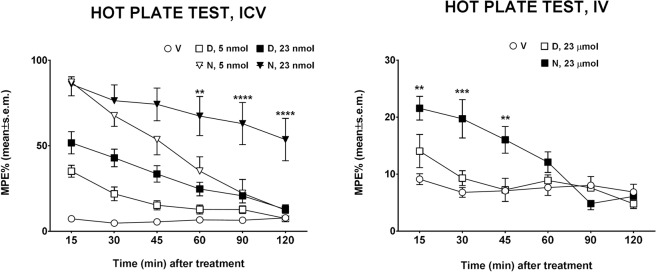
Effects induced by **NOVA2 (N)** and **DPDPE (D)** in the hot plate test in mice. In the left panel, the effects induced by i.c.v. administered **NOVA2** and **DPDPE** at doses of 5 and 23 nmol are reported. In the right panel, the effects induced by i.v. administered **NOVA2** and **DPDPE** at the dose of 23 μmol are reported. ** is for P < 0.01, *** is for P < 0.001 and **** is for P < 0.0001 comparing **NOVA2** *vs* **DPDPE** at the same dose. N = 6–9.

The antinociceptive activity of **NOVA2** was evaluated using the hot plate and formalin test. The hot plate test is frequently used for evaluating thermal pain sensitivity as a rapid and precise screening of presumptive anti-nociceptive efficacy of new compounds on murine laboratory species. The hot plate test evaluates animal pain sensitivity before and after treatment, measuring thermal pain reflexes due to footpad contact with a heated surface. **NOVA2** was able to increase nociceptive threshold after administration in the cerebral ventricle of mice (Fig. 5). The antinociceptive effects of **NOVA2** were observed both after low (5 nmol) and high (23 nmol) doses. **NOVA2** administered at the dose of 5 nmol induced a small increase in %MPE in comparison to DPDPE-treated animals at the same dose, but statistical analysis did not show significant differences when comparing the groups. When **NOVA2** was administered at the dose of 23 nmol, it induced a long-lasting antinociceptive effect in comparison to DPDPE-treated animals, that was statistically significant from 60 to 120 min after central administration. Furthermore, **NOVA2** administered intravenously at the dose of 23 μmol^.^kg^−1^ was able to increase nociceptive threshold from 15 to 45 min after injection while DPDPE was ineffective when given by this route to modify the response to thermal stimuli (Fig. 5).

**Figure 6:**
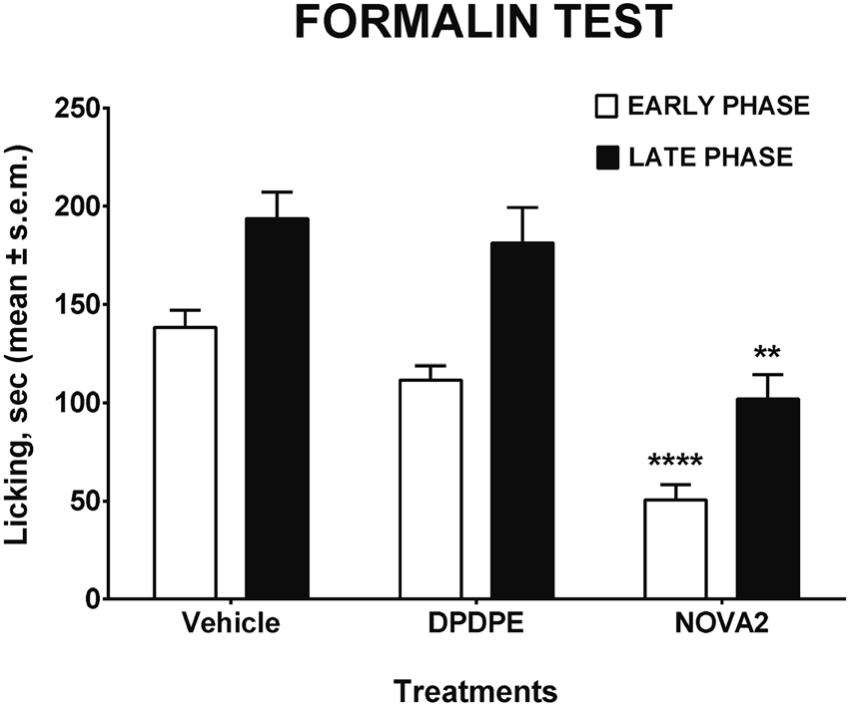
Effects induced by s.c. administered **NOVA2** and **DPDPE** in the formalin test in mice. **DPDPE** and **NOVA2** were administered at the dose of 100 nmol. **** is for P < 0.0001 and ** is for P < 0.01 *vs* DPDPE. N = 8.

The formalin test involves moderate and continuous pain generated by an injured tissue, and the measured response is the time the animals spend licking the injected paw. The behavioral response to formalin shows an early and a late phase. The early phase is caused predominantly by δ-fiber activation, due to a direct effect on nociceptors, while the late phase appears to be an inflammatory response, involving the unmyelinated C-fibre^44^. In the formalin test, s.c. **NOVA2** induced a significant antinociceptive effect, observed both in the early and in the late phase of the test. In comparison, DPDPE after s.c. administration induced a slight but not significant antinociceptive effect (Fig. 6). These results confirm the long-lasting antinociceptive efficacy of **NOVA2**, that was able to increase nociceptive threshold both after central (hot plate test) and peripheral (formalin test) administration in mice.

The exact mechanism for this effect is unclear at present, but some hypotheses can be formulated. The cyclization of peptides reduces the molecular conformational freedom and generally increases metabolic stability. Due to this increased stability, cyclized peptides might stimulate target receptors for a longer time and diffuse to other brain areas near the injection site involved in pain control, thus exerting a more pronounced antinociceptive effect then the parent compounds. In several cases, peptide analogues have been found to possess much higher biological activity than that expected on the simple basis of binding studies or tissue bio-assays^45^. Furthermore, intravenous administration of **NOVA2** to mice resulted in antinociception in the hot plate test, indicating that **NOVA2** was able to permeate the BBB.

### Molecular docking

A computational approach was employed to explore the interaction mode on the MOP and DOP of the triazole ring incorporated in the novel compounds **NOVA1** and **NOVA2**. The docking of the novel molecules compared with crystallographic ligands and DPDPE was performed on the crystal structures of the MOP (5C1M) and DOP (4RWD) downloaded from the PDB database and prepared by the PrepWizard module embedded in Maestro 2017^46–48^. The missing side chains were added, and all the co-crystallized molecules were removed from the receptors, except for the ligand and the water molecules 1303 and 101 for DOP and 526 and 538 for MOP. These water molecules appear to be involved in a water network connecting the ligand to a key residue of Histidine in both cases.

This hydrogen bond network involving the OH phenolic group of the ligand, two water molecules, His278 for MOP, and His297 for DOP, was optimized by the Interactive H-bond Optimizer interface, contained in the PrepWizard module. This tool is able to improve the orientation of the hydrogens by automatically finding the best possible interactions. After this optimization, the water network for both receptors was well defined (see Fig. S11, SI) and seems to involve hydrogen bonds to the backbone of the surrounding residues Lys233 and Lys214 for MOP and DOP, respectively.

After this step, the grid (suitable for peptides) was generated by Glide, and the water molecules were also included^49^. Several grids were generated and calculated around the crystallographic ligands in a box sized from 10 to 30 Å. The crystallographic ligands were docked to the respective receptors by using the above generated grids in order to validate docking methods and the best scoring functions. However, Glide was only able to return an acceptable pose of the crystallographic ligand for the MOP, whereas in the case of DOP, the self-docking was not satisfying; the docking returned a pose with RMSD always greater than 5 Å, even after using different docking parameters, grid sizes, and scoring functions.

Following a previously reported paper by Schuster *et al*., the docking software Gold was tested for validation^50^. At this stage, the software GOLD 6.0 was configured for self-docking involving the crystallographic MOP/DOP ligand-receptor complexes as described above. The two water molecules connecting the hydroxyl group of the tyrosine-like portion of the crystallographic ligands to His297 in MOP and His278 in DOP were also inserted in the docking parameters configuration file of GOLD. All the scoring functions of GOLD (ASP, PLP, GOLDSCORE and CHEMSCORE) were considered to conduct the validation tests, performing a self-docking of the crystallographic ligands, and by comparing the RMSD of the best docked crystallographic pose with the original one by Glide. The self-docking was performed in the presence of the water molecules, following a well validated procedure previously reported for the MOP (PDB id:4DKL) by Schuster *et al*.^50^ The two water molecules were set to “toggle and spin”, in order to allow the program to automatically decide whether or not the water molecules should be included during the docking and to optimize their orientation. An area of 10 Å around the co-crystallized ligand was defined as the binding site.

At the end of the docking calculations, the GoldScore scoring function returned with the lowest RMSD values (Table S2, see SI). The GoldScore fitness function also demonstrated a positive involvement of the water molecules in the re-building of the crystallographic water network to His297 and His278. The RMSD value of 2.33 Å for the self-docking on MOP might appear high at first sight, however, it must be considered that the pseudo-tetrapeptide nature of its crystallographic ligand, possessing rotatable bonds, implies a high conformational variability (Fig. S12, see SI).

**Figure 7:**
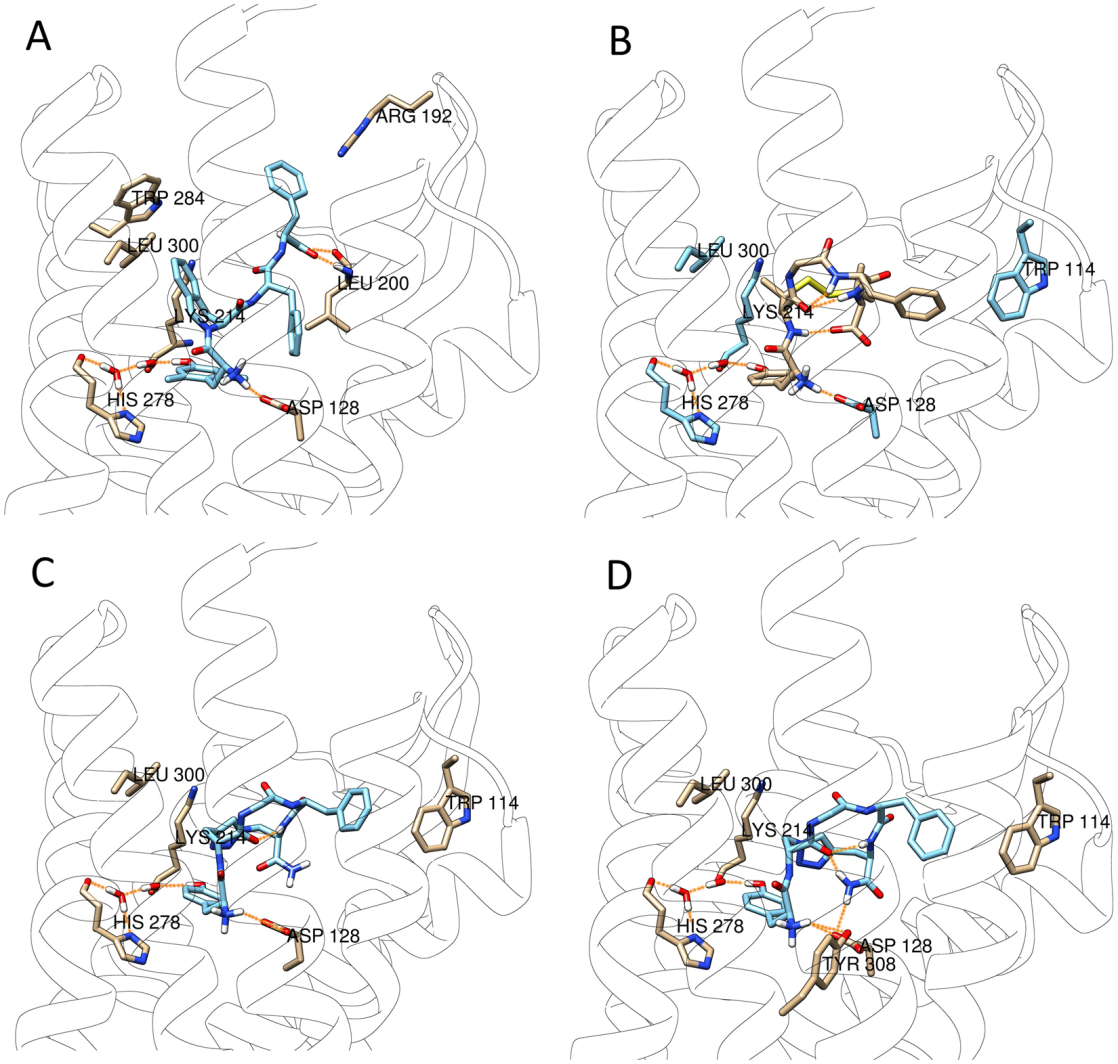
Best ranked docking poses of TIPP-NH_2_ (**A**), DPDPE (**B**), **NOVA1** (**C**) and **NOVA2** (**D**) docked at the DOP (4RWD).

Following these validation studies, Gold with the GoldScore fitness function was selected for the docking study of DPDPE as a reference compound, along with **NOVA1** and **NOVA2** at both receptors 4RWD (DOP) and 5C1M (MOP). We also included the water network, due to its key role in the interaction of ligands with the MOP (PDB id: 4DKL), by mediating a polar interaction with His297^50^. These docking results are reported in Table S3 (see SI). At the DOR, **NOVA1** and **NOVA2** showed a similar interaction behavior, by assuming a convergent conformation and by establishing similar interactions to the key residues Asp128, His278 and to Trp114 and Lys214. Some of these interactions have been found with the crystallographic ligand TIPP-NH_2_. TIPP-NH_2_ interacts with residues Trp284, Leu200 and Arg192, which are missing for **NOVA1** and **NOVA2** (Fig. 7). Furthermore, DPDPE shares with **NOVA1** and **NOVA2** the interactions to Asp128 and His278 through the water molecule network and Trp114. However, both **NOVA1** and **NOVA2** appear to strongly bind to MOP and DOP but are not capable of stimulating the activation of G protein coupled to DOP. It is possible that the additional interactions found for TIPP-NH_2_ are crucial for the coupling of the DOP to G proteins. Both the docked poses of **NOVA1** and **NOVA2** present the same aromatic ring orientation of Phe^4^, comparable to that of DPDPE (Fig. S13 **A**).

Both aromatic rings of Tyr^1^ and Phe^4^ are believed to be important for biological activity, on the other hand the cyclization bridge should only have a structural role, like in DPDPE. However, in our case the triazole ring introduced additional interactions with the Lys214 side chain. This additional connection between **NOVA1** and **NOVA2** to the receptors is not possible for DPDPE, which is a full agonist of DOP. We have noticed the same behavior in our previous work in which three other models of DPDPE bearing an aromatic bridge have been synthesized and tested^26^. In this work we found that the models showing the interactions between the aromatic bridge and the Lys214 were the most inactive, whereas the model not featuring this interaction was the most active.

**Figure 8:**
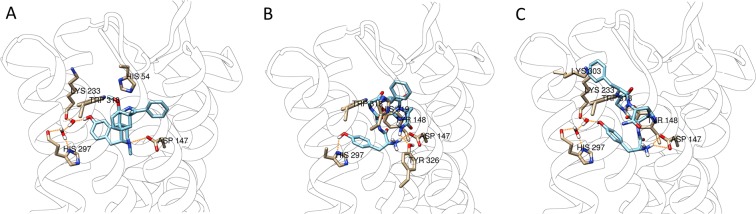
Best ranked docking poses of BU72 (**A**), **NOVA1** (**B**), **NOVA2** (**C**), docked at the ΜΟP (PDB:5C1M).

At the MOP, **NOVA1** interacts with the key residues Asp147, Tyr326 and His297 by direct hydrogen bond, and thus not mediated by the water network. Other interactions are found to His319 and Tyr148. For **NOVA2**, we found the same key interactions to Asp147, Tyr148 and His297 through the water network, but the interaction to His319 was missing, whereas the interaction to Lys303 was still present (Fig. 8). Compared to the crystallographic ligand Bu72, **NOVA1** and **NOVA2** have in common the interactions to Asp147, His297, and one relevant difference in the interaction to His54 which is present in the crystallographic ligand and missing in both **NOVA** compounds. In this regard, the residue His54 has been shown to play a role in the interaction with the crystallographic ligand, however this residue is part of a flexible loop which is not present in other crystal structures of the MOP such as 4DKL, suggesting that this residue is not crucial.

The significant differences between the binding affinity of **NOVA1** and **NOVA2** could be explained by the comparison of the side chain orientation of the Phe^4^ residue (Fig. S13 **B**). Indeed, the phenylalanine aromatic rings of the two compounds are positioned in different orientations, thus potentially reducing the entropic component in the **NOVA1**-protein interaction. Our findings revealed the presence of a similar interaction mode to DOP (4RWD) and MOP (5C1M) for both **NOVA** compounds including the crucial role of Asp147, which has been described by several groups^47,51^.

It is well accepted that there is a similar interaction present in the DOP involving Asp128. On the other hand, the interaction with the key amino acid residues Asp147 and Tyr148 on the MOP (5C1M) is well known and our ligands are able to form such interactions. Also, it is worth noting that not all the active ligands have shown the interaction to His297 either directly (**NOVA1**) or mediated by the water network (**NOVA2**), hence this interaction shouldn’t be considered as crucial.

Intriguingly, the novel compounds reported in this work are full agonists at the MOP and weak partial agonists at the DOP. This behavior is not explained by the different docking poses obtained at MOP and DOP. It can be speculated that both novel ligands bind at the DOP cavity to a key residue, such as Lys214, thus blocking the conformational change in the 4RWD structure needed for activation. Indeed, this interaction is missing in the DPDPE pose. This hypothesis is further supported by the findings of Schuster *et al*.^50^ on the base of which the interaction of some ligands with Lys233 of the MOP could prevent receptor activation. Overall these modeling experiments provide significant support to the biological data, by highlighting different modes of interaction of the novel ligands compared to the reference structures.

### Plasma stability

A plasma stability assay was performed by incubation of compounds in human plasma at a chosen initial concentration (i.e. 5 μM) at 37 °C. The aliquots of the incubated plasma samples were taken at T0 (at the beginning) and at T2 (after 4 hours). Collected samples were spiked with cold acetonitrile to precipitate proteins and prevent the compound from further decomposition. The assay was repeated in triplicate. Plasma aliquots were centrifuged and the supernatant collected and analyzed by LC-MS technique. The instrument responses were referenced to the zero time-point sample (as 100%) in order to determine the percentage of compound remaining after incubation at a given time point. The half-life parameter was calculated from linear regression of time course data. Degradation curves (Fig. S14, see SI) were plotted as the total amount of remaining parent compound (expressed as %) versus time (as minutes), revealing improved stability of **NOVA2** compared to eucatropine in human plasma. The degradation half-life (t_1/2_) of **NOVA2** was obtained by least-squares linear regression analysis of peptide pick area versus time and found to be >240 min. These results confirm that **NOVA2** possesses enhanced metabolic stability, which could explain in part its efficacious activity with peripheral administration in mice.

### BBB PAMPA assay

The blood brain barrier permeability of **NOVA2** was predicted through a parallel artificial membrane permeability assay (PAMPA), first described by Kansy *et al*.^52^. Porcine brain lipids were used to estimate BBB penetration (see SI). The ability to permeate the artificial membranes was classified according to the literature^53^, as:

$${\rm{Pe}} > 4.0\to {\rm{high}}\,{\rm{permeation}},$$

$${\rm{Pe}} < 2.0\to {\rm{low}}\,{\rm{permeation}}.$$

The lipophilicity of a peptide, the number of potential hydrogen bonds, the conformational structure and molecular size are all key factors responsible for passive transport across the bilayer membrane. DPDPE has a low lipophilicity and a very low tendency to pass the BBB by passive penetration, as demonstrated by the neutral phospholipid bilayer membranes model. In contrast, the linear analogue DPDPE(SH)_2_ shows an increased permeability (Pe average value 4-fold higher than that observed for DPDPE), which may be correlated to its ability to undergo conformational changes at the membrane surface^54^. A previously reported NMR study demonstrated very little capacity for DPDPE to change conformation upon interaction with lipid bilayers, due to the well-defined β-turn structure in solution that makes difficult to shed water on hydration^55^. **NOVA2** exhibits a low permeability across the BBB, however its Pe value is two-fold higher than that of the negative control theophylline. **NOVA2** is able to cross the membrane better than the fluorinated cyclopeptide Dmt-*c*[D-Lys-Phe-p-CF_3_-Phe-Asp]NH_2_ (**F18**) in the PAMPA assay (Table 1)^56^, despite the presence of Tyr^1^ residue (in place of Dmt^1^) which increased the number of potential hydrogen bonds, decreasing lipophilicity and a natural Phe^4^ in place of p-CF_3_-Phe^4^.

**Table 1:** Blood-brain barrier permeability (PAMPA) results for **NOVA2**.

Compounds	BBB permeability Pe (cm^.^s^−1^)	Mean (%) Mass balance	Classification
Verapamil	333.80^.^10^–7^	97.16	High permeation
Theophylline	<5.87^.^10^–7^	99.38	Low permeation
**NOVA2**	<11.93^.^10^–7^	102.22	Low permeation
**F18**: Dmt-*c*[D-Lys-Phe-p-CF_3_-Phe-Asp]NH_2_^56^	<5.8^.^10^–7^	95.75	Low permeation

As in the case of DPDPE-containing disulphide bridge, the incorporation of the triazole moiety reduces the conformational flexibility of the peptide, making it difficult to interact with the membrane surface and forcing the exposure of the hydrophobic surface determined by aromatic side chains of Tyr and Phe to the solvent solution. However, we cannot exclude the involvement of carrier-mediated transport^57,58^, and endocytic mechanisms at the BBB surface. The antinociceptive effect of peripherally administered opioid ligands depends on their metabolic stability and ability to cross the BBB; **NOVA2** is stable in human plasma and showed low permeation in the BBB-PAMPA model. However, an intense antinociceptive response could be observed after s.c. administration, confirming the capacity of our novel compound to induce antinociceptive effects, either at a peripheral or central site of action.

## Conclusion

In summary, we have discovered two new DPDPE analogues via solid-phase synthesis of two enkephalin precursors and their cyclization by Cu^I^-catalyzed azide-alkyne cycloaddition. This straightforward methodology represents a viable synthetic alternative to the previously established DPDPE cyclization^20–23,26^. This methodology also permits us to probe the feasibility of the click chemistry approach and to test the impact of these modifications on the biological activities of these analogues. The strengths of this reaction consist of wide functional group compatibility, mild reaction conditions, regioselective formation of the 1,4-disubstituted isomer, good yields, and ease of product purification^28,30^. The click reaction for compounds **NOVA1** and **NOVA2** proceeded smoothly in each case, employing CuBr as the Cu(I) catalyst. Products were obtained in good yields after simple cleavage and RP-HPLC purification. An efficient synthetic methodology has been probed to readily afford these cyclic peptides via SPPS on resin *side chain to side chain* cyclization involving a CuAAC reaction leading to the formation of a triazole bridge, a useful tool to constrain peptides^59^.

The cyclization of the enkephalin chain by triazole-containing moieties improved affinity to the MOP, whereas the compounds have extremely low affinity towards the KOP. These analogues further enhance selectivity for the MOP vs. the DOP, opposite from the DPDPE parent, with apparent high intrinsic efficacy at the MOP with **NOVA2** displaying the most potent (12.9 nM) activity. This activity was reflected in a highly efficacious and long-lasting antinociceptive effect *in vivo* by the hot plate test after i.c.v. administration and the formalin test after s.c. administration for **NOVA2**. Owing to its redox stability and dissimilarity to common natural building blocks, improved pharmacokinetic properties in plasma were observed for this disulphide surrogate, which may explain the long-lasting antinociceptive activity, well beyond that of DPDPE.

Despite **NOVA2** demonstrating a low permeability to the BBB in the PAMPA assay, it was metabolically stable in human plasma, showing a t_1/2_ >240 min and an anti-nociceptive effect after i.v. administration significantly higher than that of DPDPE^60^. This cyclization strategy may help to overcome the difficulties that often arise during oxidative folding of cysteine rich peptides *in vitro*. The straightforward introduction of azide and alkyne moieties into structurally diverse peptide side chains, combined with optimized on-resin macrocyclization conditions, will facilitate the general application of triazoles in the design of structurally constrained peptides. These novel chemical entities may lead to the development of therapeutic compounds for use in the treatment of pain. This synthetic approach promises to further expand the repertoire of compounds to be explored that target the opioid receptors.

## Experimental Section

### Chemical synthesis

HPLC grade solvents were purchased from VWR International (Milano, MI); Tentagel-S-NH_2_ resin, HATU, and all Fmoc-protected amino acids were purchased from IRIS Biotech (Marktredwitz, DH); biochemical grade trifluoroacetic acid for HPLC was acquired from VWR International (Milano, MI), and standard grade trifluoroacetic acid for deprotection of peptides was purchased from Sigma Aldrich (Milano, MI). DMSO-d_6_ was acquired from Cambridge Isotopes (Massachusetts, MA); all other reagents were from Sigma Aldrich (Milano, MI). Final products were purified by RP-HPLC using a Waters XBridge BEH130 C18, 5.0 μm, 250 mm × 10 mm column at a flow rate of 4 mL.min^−1^ on a Waters Binary pump 2996, using as eluent a linear gradient of H_2_O/acetonitrile 0.1% TFA ranging from 5% acetonitrile to 90% acetonitrile in 32 min. The purity and the retention time (R_t_) have been established by analytical UPLC-MS (C18-bonded 4.6 mm × 150 mm) at a flow rate of 1 mL^.^min^−1^, using as eluent a gradient of H_2_O/acetonitrile 0.1% TFA ranging from 10% acetonitrile to 90% acetonitrile in 20 min and was found to be ≥ 95%. UV detection (214 nm) was chosen for semipreparative HPLC; the novel chemical entities were identified with ESI-HRMS and ^1^H-NMR spectroscopy. ^1^H-NMR and 2D TOCSY-NMR spectra were performed in DMSO-d_6_ solution on a Varian Mercury operating at the 1 H frequency of 300 MHz (Figs S7–S10, see SI). Chemical shifts were referred to the residual proton signal of DMSO at 2.5 ppm. HRMS was performed using an Q Exactive Hybrid-Quadrupole Orbitrap mass spectrometer.

H-Tyr-*c*[DPra-Gly-Phe-DAza]-NH_2_ triazole (**NOVA1**). 15% overall yield. R_t_ (HPLC) = 13.49 min. ^1^H NMR ((CD_3_)_2_SO) δ 9.34 (s, 1 H, OH Tyr), 8.75 (d, 1 H, NH Phe), 8.63 (dd, 1 H, NH Gly), 8.42 (d, 1 H, NH DPra), 8.01 (brs, 3 H, NH_3_^+^ Tyr), 7.63, 7.37 (s, 2 H, NH_2_ amide), 7.44 (s, 1 H, 1’-H triazole), 7.34 (d, 1 H, NH DAza), 7.25–7.16 (m, 5 H, Phe ArH), 7.10 (dd, 2 H, C^2,4^H Tyr), 6.68 (dd, 2 H, C^3,5^H Tyr), 4.80 (dd, 1 H, ^β^CH DAza), 4.65 (q, 1 H, ^α^CH DPra), 4.58 (dd, 1 H, ^β^CH DAza), 4.38 (q, 1 H, ^α^CH DAza), 4.28–4.15 (m, 3 H, ^α^CH Tyr, Phe and CH_2_ Gly), 3.22 (dd, 1 H, CH_2_ Gly), 3.06–2.79 (m, 6 H, ^β^CH_2_ DPra, Tyr, Phe). HRMS (m/z): [M-H]^+^ calcd. for C_28_H_34_N_9_O_6_, 592.26320; found, 592.26311.

H-Tyr-*c*[DAza-Gly-Phe-DPra]-NH_2_ triazole (**NOVA2**). 12% overall yield. R_t_ (HPLC) = 13.29 min. ^1^H NMR ((CD_3_)_2_SO) δ 9.36 (s, 1 H, OH Tyr), 9.06–8.94 (m, 2 H, NH Gly, DPra), 8.65 (d, 1 H, NH DAza), 8.01 (brs, 3 H, NH_3_^+^ Tyr), 7.54 (s, 1 H, NH_2_ amide), 7.45 (s, 1 H, 1’-H triazole), 7.27–7.15 (m, 7 H, Phe ArH, NH_2_ amide and NH Phe), 7.13 (dd, 2 H, C^2,4^H Tyr), 6.70 (dd, 2 H, C^3,5^H Tyr), 4.84 (q, 1 H, ^α^CH DAza), 4.53 (dd, 1 H, ^β^CH DAza), 4.42–4.34 (m, 2 H, ^α^CH Phe, ^β^CH_2_ DAza), 4.27–4.17 (m, 3 H, ^α^CH Tyr, DPra, CH_2_ Gly), 3.43–3.30 (m, 1 H, CH_2_ Gly under water), 3.18–2.97 (m, 4 H, ^β^CH_2_ DPra, Phe, Tyr), 2.86–2.77 (m, 2 H, ^β^CH_2_ DPra, Tyr). HRMS (m/z): [M-H]^+^ calcd. for C_28_H_34_N_9_O_6_, 592.26320; found, 592.26326.

### Cell lines and cell culture

Chinese hamster ovary (CHO) cells expressing the human MOP, DOP, or KOP were used for all experiments. The details for these cell lines, including their K_D_ values for ^3^H-diprenorphine binding, can be found in Stefanucci *et al*.^61^ The cells were maintained in 1:1 DMEM/F12 culture media (Gibco), with 1X penicillin/streptomycin, and 10% heat-inactivated fetal bovine serum (Gibco) in a 5% CO_2_ atmosphere 37 °C incubator. For experiments, cells were harvested using 5 mM EDTA in PBS, collected, centrifuged, and stored at −80 °C. Membrane preparations for binding or GTPγS coupling were created using the same protocol as reported^61^.

### Competition radioligand binding

Competition radioligand binding was performed exactly as reported in Stefanucci *et al*.^61^. Membrane preparations of receptor-containing CHO cells were combined with concentration curves of **NOVA** compounds or positive control, and a fixed concentration (4.33–5.25 nM) of ^3^H-diprenorphine (PerkinElmer). Vehicle concentrations were equalized between each reaction. Reactions were incubated for 1 hour at room temperature. The resulting plates were read using a PerkinElmer MicroBeta2 6-detector 96-well format scintillation counter. The data was normalized to ^3^H-diprenorphine alone (100%) or non-specific binding measured by the inclusion of 10 μM naloxone (0%). IC_50_ values for each curve and the calculated K_i_ using the established K_D_ of ^3^H-diprenorphine in each cell line was calculated using GraphPad Prism 7.0, and reported as the mean ± SEM.

### ^35^S-GTPγS coupling assay

The GTPγS assay was again performed exactly as reported in Stefanucci *et al*.^61^ Membrane preparations of receptor-containing CHO cells were combined with concentration curves of **NOVA** compound or positive control along with 0.1 nM ^35^S-GTPγS (PerkinElmer), and incubated at room temperature for 1 hour. Vehicle concentrations were normalized between each reaction. The resulting plates were read as above, and the data normalized to the stimulation caused by positive control compound (100%) or vehicle (0%). The potency (EC_50_) and efficacy (E_Max_) values were calculated for each curve using GraphPad Prism 7.0 and reported as the mean ± SEM. The efficacy was defined for each compound in relation to the maximum efficacy of the positive control compound, defined as 100%.

### Animals and ethical statement

CD-1 male mice (Harlan, Italy) weighing 25–30 g were used in all the experiments. Before the experimental sessions, the mice were maintained in colony, housed in cages (7 mice per cage) under standard light/dark cycle (from 7:00 AM to 7:00 PM), temperature (21 ± 1 °C) and relative humidity (60 ± 10%) for at least 1 week. Food and water were available *ad libitum*. The research protocol was approved by the Service for Biotechnology and Animal Welfare of the Istituto Superiore di Sanità and authorized by the Italian Ministry of Health, according to Legislative Decree 26/14, which implemented the European Directive 2010/63/UE on the protection of laboratory animals in Italy. Animal welfare was routinely checked by veterinarians from the Service for Biotechnology and Animal Welfare. Animal studies are reported in compliance with the ARRIVE guidelines^62,63^.

### Hot plate test

Thermal nociception (hot plate test) was assessed with a commercially available apparatus consisting of a metal plate 25 × 25 cm (Ugo Basile, Italy) heated to a constant temperature of 55.0 ± 0.1 °C, on which a plastic cylinder (20 cm diameter, 18 cm high) was placed. The time of latency (s) was recorded from the moment the animal was placed in the cylinder on the hot plate until it licked its paws or jumped; the cut-off time was 60 s. The baseline was calculated as mean of three readings recorded before testing at intervals of 15 min. The time course of latency was then determined at 15, 30, 45, 60, 90 and 120 min after compound treatment. Data were elaborated as time-course curve of the percentage of maximum effect (%MPE) = (post drug latency – baseline latency)/(cut-off time – baseline latency) × 100. In these experiments, compounds were administered by intracerebroventricular (i.c.v.) or intravenous (i.v.) injections. For i.c.v. injections, mice were lightly anesthetized with isoflurane, and an incision was made in the scalp. Injections were performed using a 10 μL Hamilton microsyringe at a point 2-mm caudal and 2-mm lateral from the bregma at a depth of 3 mm in a volume of 10 μL as previously described^64^.

### Formalin test

Subcutaneous injection of a dilute solution of formalin (1%, 20 μL/paw) into the mice hind paw evoked nociceptive behavioural responses, such as licking, biting the injected paw or both, which are considered indices of nociception^65^. The nociceptive response showed a biphasic trend: an early phase, occurring from 0 to 10 min after formalin injection, produced by the direct stimulation of peripheral nociceptors, and a late prolonged phase, occurring from 15 to 40 min, which reflected the response to inflammatory pain. During the test, the mouse was placed in a Plexiglas observation cage (30 × 14 × 12 cm), 1 h before the formalin administration and allowed to acclimatize to the testing environment. The total time the animal spent licking or biting its paw during the early and late phase of formalin-induced nociception was recorded. In these experiments, compounds under investigation were administered subcutaneously (s.c.) into the mice in a volume of 20 μL/mouse 15 min before formalin.

### Data analysis and statistics

Experimental data were expressed as mean ± SEM. Significant differences among the groups were evaluated with an analysis of variance (ANOVA) followed by Tukey’s post-hoc comparisons using the GraphPad Prism 6.03 software. Statistical significance was set at P < 0.05. The data and statistical analysis comply with the recommendations on experimental design and analysis in pharmacology^66^.

### Plasma stability method

Plasma and working solutions of experimental and control compounds (at appropriate concentrations) were warmed to 37 °C. 99 µL of plasma was dispensed into a pre-labeled 96-well plate (in triplicate per time-point). Then 1 µL of test compound and reference compound were transferred to wells filled by plasma. 200 µL of cold acetonitrile was added immediately into wells marked as the ‘0’ time point to precipitate proteins and prevent compound from biotransformation. Plates were incubated in a thermostatic shaker at 37 °C while shaken at 350 rpm. The reaction was quenched by the addition of 200 µL of cold acetonitrile to appropriate wells at predetermined time points (50, 100, 150, 200, 250 min). After the last time point, the plate was centrifuged at 4000 × g for 20 minutes at 4 °C. 200 µL of supernatant was transferred to 96-well plates and covered with plate mats. Samples were analyzed using an LC-MS technique.

### PAMPA assay method

Donor: acceptor solutions of experimental and reference compounds (concentration 10 mM) were diluted with a mixture of PBS-buffered saline (pH 7.4) and ethanol (30%) to a final concentration of 200 μM. Acceptor solutions were prepared by mixing PBS/EtOH (30%) with 2% DMSO. A 1% solution of porcine polar brain lipids was prepared by dissolving an appropriate amount of PPBL in dodecane. Sonication of the lipid/organic solvent solution was performed to insure complete dissolution of the lipid.

## Supplementary information

cover letter
