# Supplementary material for: On resin click-chemistry-mediated synthesis of novel enkephalin analogues with potent anti-nociceptive activity
Source: Sci Rep. 2019 Apr 8;9:5771. doi: 10.1038/s41598-019-42289-5 (PMC6453917; doi:10.1038/s41598-019-42289-5)
Supplement: Supplementary file 1 — cover letter [file 41598_2019_42289_MOESM1_ESM.docx]

**SUPPORTING INFORMATION**

**ON RESIN CLICK-CHEMISTRY-MEDIATED SYNTHESIS OF NOVEL ENKEPHALIN ANALOGUES WITH POTENT ANTI-NOCICEPTIVE ACTIVITY**

Azzurra Stefanucci,^1^ Wei Lei,^2^ Stefano Pieretti,^3^ Ettore Novellino,^4^ Marilisa Pia Dimmito,^1^ Francesca Marzoli,^3^ John M. Streicher,^2^ and Adriano Mollica^1,*^

^1^Dipartimento di Farmacia, Università di Chieti-Pescara “G. d’Annunzio”, Via dei Vestini 31, 66100 Chieti, Italy.

^2^Department of Pharmacology, College of Medicine, University of Arizona, Tucson, AZ USA.

^3^Istituto Superiore di Sanità, Centro Nazionale Ricerca e Valutazione Preclinica e Clinica dei farmaci, Viale Regina Elena 299, 00161 Rome, Italy.

^4^Dipartimento di Farmacia, Università di Napoli “Federico II”, Via D. Montesano, 49, 80131 Naples, Italy.

*^*^ Corresponding Author*

[a.mollica@unich.it](mailto:a.mollica@unich.it)

| **Supporting Information** | |
| --- | --- |
| *Table of contents* | |
| Fmoc-SPPS/CuAAC synthetic protocol | S2,S3 |
| Figure S1 | S4 |
| Figure S2 | S5 |
| Figure S3 | S6 |
| Figure S4 | S8 |
| Figure S5,S6 | S9 |
| Figure S7 | S10 |
| Figure S8 | S11 |
| Figure S9 | S12 |
| Figure S10 | S13 |
| Table S1 | S14 |
| Table S2, Figures S11, S12 | S15 |
| Table S3 | S16 |
| Figure S13 | S17 |
| Figure S14 | S18 |
| **BBB PAMPA** procedure | S19 |

**Fmoc-SPPS/CuAAC synthetic protocol**

Tentagel-S-NH_2_ resin (384.0 mg, 0.26 mmol/g) was transferred into a peptide synthesis plastic vessel; the resin was suspended in 5 mL of dry CH_2_Cl_2_ for 40 min. A solution of Rink-amide linker (215.8 mg, 0.41 mmol), TBTU (128.4 mg, 0.41 mmol), HOBt anhydrous (54.1 mg, 0.41 mmol) in 7 mL DMF and DIPEA (0.8 equiv., 0.14 mL), was prepared and added to resin shaked for 12 hours. The resin was drained, washed 3 times with DMF, 3 times with CH_2_Cl_2_ (5 mL each) and treated 2 times with 1:1:5 acetic anhydride/DIPEA/CH_2_Cl_2_ mixture (6 min per treatment), shaking to cap unreacted sites. The resin was then washed with DMF (5 mL) 3 times, CH_2_Cl_2_ (5 mL) 3 times.

**Peptide elongation.** The resin was drained with air and then submitted to cycles of manual Fmoc-solid phase peptide synthesis using amino acid building blocks: FmocTyr(*t*Bu)-OH, FmocGly-OH, FmocPhe-OH, Fmoc-DAza-OH and Fmoc-DPra-OH. Each step consisted of Fmoc-deprotection with 20% piperidine in DMF, washing with DMF and DCM. Coupling of Fmoc-DAza-OH and Fmoc-DPra-OH (0.41 mmol, 4 equiv.) was performed in a mixture of HATU (0.41 mmol, 4 equiv.) and DIPEA (5 equiv., 0.09 mL) in DMF at r.t. overnight, while a mixture of TBTU (0.41 mmol, 4 equiv.), HOBt anhydrous (0.41 mmol, 4 equiv.) and DIPEA (0.8 mmol, 0.14 mL) in DMF at r.t., was used for FmocGly-OH, FmocTyr(*t*Bu)-OH and FmocPhe-OH (0.41 mmol, 4 equiv.) stirring overnight. Each coupling reaction has been repeated two times and checked with Kaiser test.

**CuAAC reaction procedure on resin.** 384 mg of resin was swelled in DCM for 10 min in the plastic syringe. After the removal of DCM, CuBr (14.3 mg, 0.11 mmol) was dissolved in DMSO anhydrous (6 mL) previously bubbled with N_2_ gas for 10 min, sodium ascorbate (19.8 mg, 0.11 mmol) was dissolved in water (1 mL), DIPEA (1 mmol, 0.17 mL) and 2,6-lutidine (1 mmol, 0.12 mL) were added to the resin. Nitrogen was purged through the reaction vessel for 5 min, and gently agitated on rotary plate at r.t. for 16 h. Resin was washed with Isoproponal:DMSO (5:3, 3 mL x 3), followed by DMF (3 mL x 3) and DCM (3 mL x 3). The resin was then dried with air before deprotection and cleavage.

**Cleavage of the cyclic peptide from the resin and purification.** The resin was washed with CH_2_Cl_2_ (5 mL) 3 times. The Fmoc group was removed from the peptide, then the resin was deprotected with a solution of TFA (90.0%), DCM (5%), TIS (2.5%), and water (2.5%) (5 mL), stirring for 2 h at room temperature. The solution was collected in 250 mL round bottom flask, the resin was washed with CH_2_Cl_2_ (5 mL) 4 times and concentrated in rotary evaporator. The crude peptide was precipitated with cold ethyl ether, centrifuged and washed 3 times. The white solid product was separated from liquid layer and dried in high vacuum; 1 mg of crude peptide was dissolved in 1 mL CH_3_OH for UPLC-MS analytical trace and mass spectra. The residue was purified by RP-HPLC (gradient of 5%-90% CH_3_CN, 32 min). Lyophilization of the pure fractions yielded the desired peptide as white fluffy solid in 15% and 12% theoretical yields respectively for **NOVA1** and **NOVA2**. The purity of peptides was determined by analytical UPLC-MS and was found to be ≥ 95% (linear gradient 10% to 90% acetonitrile in 20 min).

**Figure S1.** UPLC-MS of the crude linear peptide H-Tyr-DPra-Gly-Phe-DAza-NH_2_

RP-UPLC: C18 linear gradient of H_2_O/acetonitrile 0.1% TFA starting from 10% acetonitrile to 90% acetonitrile in 20 min.

**Figure S2.** UPLC-MS of the mixture of crude linear peptide H-Tyr-DPra-Gly-Phe-DAza-NH_2_ and crude product **7a (NOVA1)**. RP-UPLC: C18 linear gradient of H_2_O/acetonitrile 0.1% TFA starting from 10% acetonitrile to 90% acetonitrile in 20 min.

**Figure S3.** UPLC-MS of the purified product **NOVA1 (7a)**. RP-UPLC: C18 linear gradient of H_2_O/acetonitrile 0.1% TFA starting from 10% acetonitrile to 90% acetonitrile in 20 min.

**Figure S4.** UPLC-MS of the purified product **NOVA2 (7b)**. RP-UPLC: C18 linear gradient of H_2_O/acetonitrile 0.1% TFA starting from 10% acetonitrile to 90% acetonitrile in 20 min.

**Figure S5.** HRMS for **NOVA1**.

**Figure S6.** HRMS for **NOVA2**.

**Table of analytical data**

|  | **Rt***  **(min)** | **Neutral formula** | **m/z expected** | **m/z measured**  [M+H] | **difference** | **Error (ppm)** |
| --- | --- | --- | --- | --- | --- | --- |
| **NOVA1** | 2.57 | C_28_H_33_N_9_O_6_ | 592.26320 | 592.26311 | 0.00001 | < 5 ppm |
| **NOVA2** | 3.99 | C_28_H_33_N_9_O_6_ | 592.26320 | 592.26326 | 0.00006 | < 5 ppm |

* analytical RP-UPLC: C18 linear gradient of H_2_O/acetonitrile 0.1% TFA starting from 10% acetonitrile to 90% acetonitrile in 20 min, λ = 214 nm.

**Figure S7. ^1^H-NMR spectra of NOVA 1**

**Figure S8. TOCSY spectra of NOVA 1**

**Figure S9. ^1^H-NMR spectra of NOVA 2**

**Figure S10. TOCSY spectra of NOVA 2**

**Table S1.** ^1^H-NMR signals of cyclic peptides **NOVA1** and **NOVA2** recorded by 300 MHz Varian Mercury in DMSO-d*_6_* solvent.

| **δ (ppm)** | **NOVA1** | **NOVA2** |
| --- | --- | --- |
| OH Tyr | s, 1H, 9.34 | s, 1H, 9.36 |
| NH_3_^+^ Tyr | brs, 3H, 8.01 | brs, 3H, 8.01 |
| NH DAza | d, 1H, 7.34; J = 7.2Hz | d, 1H, 8.65; J = 6.9Hz |
| NH Gly | dd, 1H, 8.63 | m, 1H, 9.06-8.94 |
| NH Phe | d, 1H, 8.75, J = 7.2Hz | m, 1H, 7.27-7.15 |
| NH DPra | d, 1H, 8.42, J = 7.5Hz | m, 1H, 9.06-8.94 |
| Phe ArH | m, 5H, 7.25-7.16 | m, 5H, 7.27-7.15 |
| Tyr ArH | dd, 2H, 7.10, J = 8.7Hz  dd, 2H, 6.68, J =8.1Hz | dd, 2H, 7.13, J = 8.7Hz  dd, 2H, 6.70, J =9.0Hz |
| 1’-H triazole | s, 1H, 7.44 | s, 1H, 7.45 |
| NH_2_ amide | s, 1H, 7.63  s, 1H, 7.37 | s, 1H, 7.54  m, 1H, 7.27-7.15 |
| CH^α^ Tyr | m, 1H, 4.28-4.15 | m, 1H, 4.27-4.17 |
| CH^α^ Phe | m, 1H, 4.28-4.15 | m, 1H, 4.42-4.34 |
| CH Gly | m, 1H, 4.28-4.15  dd, 1H, 3.22 | m, 1H, 4.27-4.17  m, 1H, 3.43-3.30 under water |
| CH^α^ DAza | q, 1H, 4.38 | q, 1H, 4.84 |
| CH^α^ DPra | q, 1H, 4.65 | m, 1H, 4.27-4.17 |
| CH^β^ Tyr | m, 2H, 3.06-2.79 | m, 1H, 2.86-2.77  m, 1H, 3.18-2.97 |
| CH^β^ Phe | m, 2H, 3.06-2.79 | m, 2H, 3.18-2.97 |
| CH^β^ DAza | dd, 1H, 4.80; dd, 1H, 4.58;  J_AB_ = 14Hz, J_BX_ = 11.1Hz, J_AX_ = 5.4Hz | dd, 1H, 4.53  m, 1H, 4.42-4.34 |
| CH^β^ DPra | m, 2H, 3.06-2.79 | m, 1H, 2.86-2.77  m, 1H, 3.18-2.97 |

**Figure S11.** Water Networks for MOP (**A**) and DOP (**B**).

**Table S2.** Validation of GoldScore scoring function by self-docking.

| Ligands | Receptors | Water Network | RMSD (Å) of the self-docking |
| --- | --- | --- | --- |
| **TIPP-NH_2_** | 4RWD (DOP) | yes | 2.33 |
| **Bu72** | 5C1M (MOP) | yes | 0.59 |

**Figure S12.** Bu72 (**A**) and TIPP-NH_2_ (**B**) docking poses superimposed to their crystallographic ones.

**Table S3.** Interactions of DPDPE, BU72, TIPP-NH_2_, **NOVA1**, **NOVA2** in the MOP and DOP binding pockets.

| DOP interactions | | | | | | | | |
| --- | --- | --- | --- | --- | --- | --- | --- | --- |
| Compounds | Asp128 | Tyr308 | His278 | Lys214 | Trp114 | Trp284 | Leu200 | Arg192 |
| TIPP-NH_2_ | H bond | -- | H bond through water network | -- | -- | π-π | H bond | Cat-π |
| DPDPE | H bond | -- | H bond through water network | -- | π-π | -- | -- | -- |
| **NOVA1** | Ionic +  H bond | -- | H bond through water network | Cat-π | π-π | -- | -- | -- |
| **NOVA2** | Ionic +  H bond | H bond | H bond through water network | Cat-π | π-π | -- | -- | -- |
| MOP interactions | | | | | | | | |
| Compounds | Asp147 | Tyr148 | Tyr326 | His297 | His319 | Lys303 | His54 |  |
| Bu72 | H bond | -- | -- | H bond through water network | -- | -- | π-π |  |
| **NOVA1** | H bond | Cat-π | H bond | Direct H bond | π-π | -- | -- |  |
| **NOVA2** | H bond | H bond | -- | H bond through water network | -- | Cat-π | -- |  |

**Figure S13.** Superimposition of DPDPE, **NOVA1** and **NOVA2** on DOP (A), **NOVA1** and **NOVA2** on MOP (B).

**Figure S14.** Human plasma stability of **NOVA2.**

**LC-MS system description**

| Panel | Model |
| --- | --- |
| Degaser | Ultimate 3000 |
| Pump | Ultimate 3000 Pump |
| Autosampler | Ultimate 3000 RS autosampler |
| Column oven | Ultimate 3000 RS column compartment |
| Detector | Ultimate 3000 Photodiode Array Detector |
| Mass spectrometer | Bruker Daltonics amaZon Speed |

**BBB PAMPA** procedure

***PAMPA*** *assay protocol*

Investigated and control compounds were diluted in the appropriate buffer (pH =7.4) to the final concentration of 200 μM. 5 μL of PPBL solution was dispensed directly on the membrane to appropriate wells of the PAMPA donor plate. 150 μL of the compound solution was dispensed to the donor plate wells. 300 μL of the buffer at pH 7.4 was dispensed to the acceptor plate wells. Then the donor plate was inserted into the receiver plate. The whole system was agitated at RT 500 rpm for 4 hours. After that time 100 μL of samples were collected from both donor and acceptor compartments and moved to a 96 well UV-VIS transparent well for analysis.

*Measurement*

Absorbance was measured in a range 250-500 nm, interval 10 nm. Maximum of absorbance was chosen for concentration determination.

*Preparation of calibration curve*

Investigated compound was diluted in the buffer to the final concentration of 200 μM. 200 μL of this solution and 100 μL of buffer with 5% DMSO were dispensed to appropriate wells of the UV-VIS transparent plate. Calibration curve was prepared by a serial dilution of solution described above. Dilution factor -2.
